# Effectiveness of COACH2COACH and R2C2 (Relationship, Reaction, Content, Coaching) Model in Improving Peer-feedback for students’ professional development

**DOI:** 10.12669/pjms.42.3.13743

**Published:** 2026-03

**Authors:** Sana Siddiqui, Usman Mahboob, Sana Shah, Syed Muhammad Junaid

**Affiliations:** 1 Sana Siddiqui, BDS, MHPE, (PhD HPE Scholar, Khyber Medical University). Assistant Professor / Head of Department, DHPE&R, Army Medical College, Rawalpindi, Pakistan; 2 Usman Mahboob, MBBS, MPH, Doctor of HPE, FAIMER Fellow, HEA Fellow, Institute of Health Professions Education & Research, Khyber Medical University, Peshawar, Pakistan; 3 Sana Shah, BDS, MHPE, (PhD HPE Scholar, Khyber Medical University). Assistant Professor / Head of Department, Medical Education, CMH Institute of Medical Sciences Dental College, Multan, Pakistan; 4 Syed Muhammad Junaid, BDS, MSc, MHPE, Institute of Health Professions Education & Research, Khyber Medical University, Peshawar, Pakistan

**Keywords:** Coach2coach, Effectiveness, Models, Peer Feedback, R2C2

## Abstract

**Background & Objective:**

Medical students seldom receive meaningful feedback; therefore, they lack professional development skills regarding peer feedback. Although several peer feedback training interventions exist, comparative evidence demonstrating the superiority of one model over another is limited. The objective of our study was to compare two interventions, Coach2coach and R2C2 (relationship, reaction, content, coaching), for improving peer feedback for students’ professional development.

**Methodology:**

The quasi-experimental study was conducted at the Armed Forces Institute of Dentistry in 2022. The Coach2coach and R2C2 models were used for teaching peer feedback skills to final year dental students in a two-group separate sample pre-posttest design. Mini PAT was used to compare the pre-post workshop results of the Coach2coach and R2C2 models. The Kirkpatrick model guided intervention evaluation, and Wilcoxon test was used to measure the effectiveness of the Coach2coach and R2C2 models.

**Results:**

The Coach2coach model (n = 27) showed a significant change in all feedback items with significant values below 0.05 and a 60% learning gain. The R2C2 model (n = 25) also improved feedback skills, but not for items three and eight of mini-PAT, which had significant values above 0.05 with an overall learning gain of 35%. Hence, the Coach2coach model was more effective than R2C2 model in enhancing peer feedback skills.

**Conclusions:**

Both models increased students’ peer feedback abilities, allowing them to grow professionally; however, Coach2coach model was deemed more efficient.

## INTRODUCTION

Feedback serves as both an instructional instrument and a social interaction between the student and the supervisor, in the context of a respectful and trusting relationship.^1^ It is one of the most widely explored areas in medical education and has different aspects and components. One such aspect is peer feedback. The effectiveness of peer feedback is established in academic literature for improving the performance of students, achieving the goal of providing meaningful feedback in clinical training continues to be challenging.^2^ Students often voiced their dissatisfaction with various aspects of the feedback they were given, including that it was either too complicated, too slow, or too short.^3^ Peer evaluation can help healthcare students improve the quality of learning and develop reflective skills and concepts on performance standards.^4^

A review of the literature on peer assessment and feedback in higher education work emphasizes that well-structured peer feedback processes can lead to deeper learning, enhance metacognitive skills, and foster a greater sense of ownership over the educational experience.^5^ Through the introduction of individual development programs and workshops, attempts have been made to enhance the efficiency and output of peer feedback.^6^ Feedback models are fundamental as they interpret how feedback influences learning and/or how students process feedback.^7^ Two such models, that are Coach2coach and R2C2, are known for their effectiveness but have not been compared previously. The Coach2coach Model emphasizes active listening, empathy, and collaborative problem-solving to improve feedback.

Therefore, we compare the effectiveness of two structured peer-feedback models, Coach2coach and R2C2 (Relationship, Reaction, Content, Coaching), for their effectiveness in students’ professional development.

## METHODOLOGY

A six months quasi-experimental study evaluated Coach2coach and R2C2 workshops in enhancing final year dental students’ peer feedback across five domains i.e. clinical care, medical practice, teaching, patient relationships, and teamwork at AFID, Army Medical College, Rawalpindi, with participants selected irrespective of age or gender. The entire class of 52 final-year dental students was included, with no exclusions based on age or gender**.** To independently assess the effectiveness of each workshop, students were divided into two groups (Coach2coach: n = 27; R2C2: n = 25) using a lottery method to ensure an equal chance of allocation.^8^

### Ethical approval:

It was obtained from both the principal author’s scholarly institute (Ref: 1-10/IHPER/MHPE/KMU/22-43; dated June 15, 2022) and Army Medical College (Ref: 983/Trg).

To evaluate the effectiveness of the interventions, the study employed the first two levels of the Kirkpatrick model.^9^ i.e. to assesses participants’ initial reactions to the training and the extent to which participants acquired knowledge and skills as a result of the training. Pre- and post-intervention tests were administered to assess the students’ feedback abilities before and after attending the workshops.

### Dry run:

**Fig.1:**
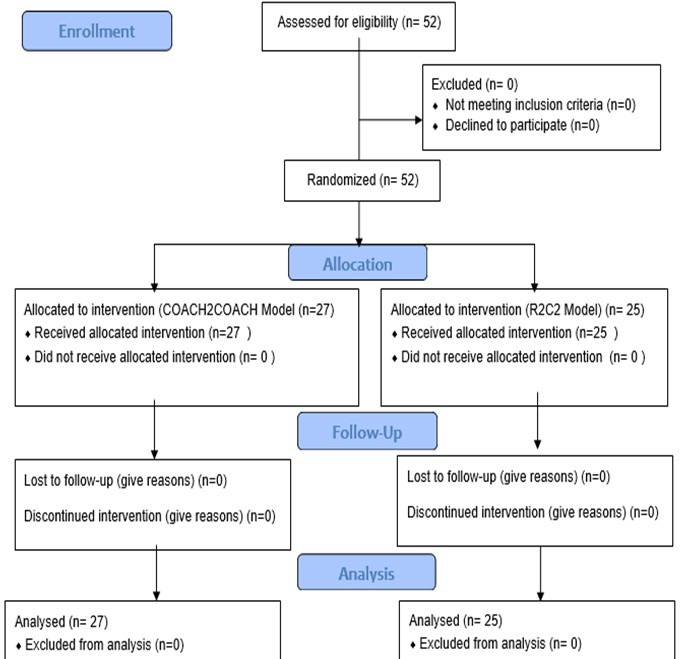
Flow chart for student selection and allocation.

A dry run was conducted two days before the workshops to ensure content validity and reliability. Facilitators tested materials and procedures, incorporated faculty feedback, and refined content and instructions. Student selection and allocation are shown in Fig.1

**Fig.2:**
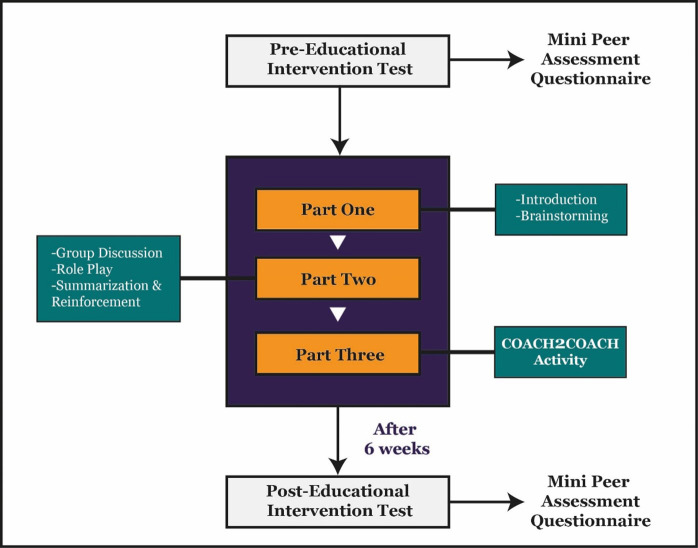
Study Intervention Procedure.

### Pre-educational intervention test:

A pre-test using the Mini-Peer Assessment Tool (Mini-PAT) was administered to all participants to assess baseline feedback skills, corresponding to Kirkpatrick Level II outcomes. The Mini-PAT comprises 16 items across three subscales: Clinical Care, Good Medical Practice, and Interprofessional Relationships.^10^ Sample items include:

- “I provide constructive feedback to my peers regarding their clinical skills.”
- “I engage effectively with my colleagues to improve patient care.”

### Peer feedback skills workshop:

Group I attended a two-hour interactive Coach2coach workshop, with participants selected by lottery and the design guided by Gagné’s Nine Principles.^11^ The session included an introduction to objectives and roles of coach and coachee, followed by role-play to enhance communication, brainstorming, and perspective-taking. Group-II attended a separate, similarly structured R2C2 workshop. All participants completed evaluation forms at the end of the sessions.

### Comparative Evaluation of Feedback Model Effectiveness Using Kirkpatrick’s Framework:

Using Kirkpatrick’s Levels I and II, participants first completed a 5 point Likert feedback form immediately after training, assessing topics covered, instructor effectiveness, presentation style, learning experience, content relevance, and overall training effectiveness. Six weeks later, the Mini-Peer Assessment Tool (Mini-PAT) evaluated Clinical Skills (diagnosis, management plans, technical skills, time/resource management, psychosocial responsiveness, and awareness of limitations), Interpersonal Skills (teaching colleagues, communication, confidentiality, recognition of contributions, accessibility/reliability), and Overall Assessment (performance compared to peers). The interval allowed participants to apply skills in clinical settings, engage in peer feedback, and demonstrate skill acquisition and retention, with pre- and post-test Mini-PAT scores enabling direct comparison of outcomes.

### Data Analysis Procedure:

IBM SPSS Statistics v25 was used for analysis. Normality was assessed with Kolmogorov-Smirnov and Shapiro-Wilk tests; since data were non-normal, quantitative variables were summarized using median and interquartile range, and qualitative variables with frequencies and percentages. Inferential analysis was performed using the Wilcoxon signed-rank test. A 100% post-test response rate was achieved, as all students were required to attend follow-up assessments.

## RESULTS

### Wilcoxon matched-pair signed-rank test:

The Wilcoxon test was employed to gauge the degree of difference in participants’ feedback as the result of the workshop based on the Coach2coach and R2C2 model. Table-I shows peer feedback on questionnaire items pre- and post Coach2coach and R2C2 workshop. Using the Barwood et al. procedure.^12^–^14^ based on the checklist, learning gains of 60% and 35% for Coach2coach and R2C2, respectively, were estimated for the participants in the program.


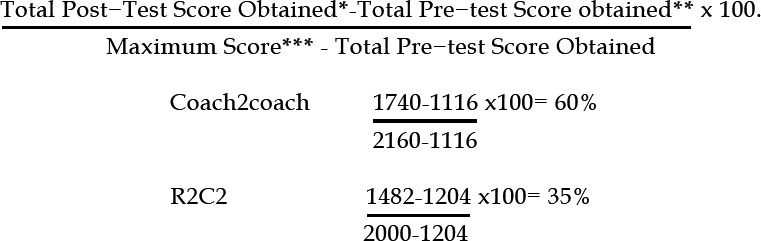


* Sum of individual checklist scores of all the participants of the posttest group

** Sum of individual checklist scores of all the participants of the pretest group

*** sum of all the items checklist scores x no of participants

**Table-I:** Peer feedback on questionnaire items pre and post Coach2coach and R2C2 workshop.

Item No.	Questionnaire Item	Workshop	Coach2coach Median (Interquartile range)	Coach2coach P-Value	R2C2 Median (Interquartile range)	R2C2 P-Value Value
1	Ability to diagnose patient problems	Pre-workshop	2.00 ± 1.00	‹ 0.001	2.00 ± 1.00	0.001
		Post-workshop	4.00 ± 3.00		3.00 ± 1.00	
2	Ability to formulate appropriate management plan	Pre-workshop	2.00 ± 1.00	‹ 0.001	2.00 ± 1.00	0.002
		Post-workshop	4.00 ± 2.00		3.00 ± 1.00	
3	Awareness of their own limitations	Pre-workshop	3.00 ± 1.00	‹ 0.001	3.00 ± 2.00	0.091
		Post-workshop	4.00 ± 2.00		1.00 ± 1.00	
4	Ability to respond to psychosocial aspects	Pre-workshop	3.00 ± 1.00	‹ 0.001	3.00 ± 1.00	0.008
		Post-workshop	4.00 ± 2.00		3.00 ± 1.00	
5	Appropriate utilization of resources	Pre-workshop	3.00 ± 1.00	‹ 0.001	3.00 ± 1.00	0.005
		Post-workshop	4.00 ± 1.00		4.00 ± 1.00	
6	Ability to manage time effectively	Pre-workshop	3.00 ± 1.00	‹ 0.001	3.00 ± 2.00	0.001
		Post-workshop	4.00 ± 2.00		4.00 ± 1.00	
7	Technical skills	Pre-workshop	2.00 ± 1.00	‹ 0.001	3.00 ± 2.00	0.013
		Post-workshop	4.00 ± 2.00		4.00 ± 1.00	
8	Willingness and effectiveness when teaching /training colleagues	Pre-workshop	3.00 ± 1.00	‹ 0.001	3.00 ± 1.50	0.444
		Post-workshop	4.00 ± 2.00		3.00 ± 1.00	
9	Communication with patients	Pre-workshop	3.00 ± 1.00	‹ 0.001	3.00 ± 1.00	0.014
		Post-workshop	4.00 ± 2.00		4.00 ± 2.00	
10	Communication with carers or/and family	Pre-workshop	3.00 ± 1.00	‹ 0.001	3.00 ± 1.00	0.009
		Post-workshop	4.00 ± 2.00		4.00 ± 1.50	
11	Respect for patients and their right to confidentiality	Pre-workshop	3.00 ± 1.00	‹ 0.001	3.00 ± 1.00	0.001
		Post-workshop	4.00 ± 1.00		4.00 ± 0.00	
12	Verbal communication with colleagues	Pre-workshop	3.00 ± 1.00	‹ 0.001	3.00 ± 0.50	0.011
		Post-workshop	4.00 ± 1.00		4.00 ± 1.00	
13	Written communication with colleagues	Pre-workshop	3.00 ± 1.00	‹ 0.001	2.00 ± 1.50	0.004
		Post-workshop	4.00 ± 2.00		4.00 ± 1.00	
14	Ability to recognize and value the contribution of others	Pre-workshop	2.00 ± 1.00	‹ 0.001	3.00 ± 2.00	0.006
		Post-workshop	4.00 ± 2.00		4.00 ± 1.00	
15	Accessibility / reliability	Pre-workshop	3.00 ± 1.00	‹ 0.001	3.00 ± 1.00	0.000
		Post-workshop	4.00 ± 2.00		4.00 ± 1.00	
16	Overall, how do you rate this doctor compared to another doctor of the same grade	Pre-workshop	3.00 ± 0.00	‹ 0.001	4.00 ± 1.00	0.001
		Post-workshop	4.00 ± 2.00		4.00 ± 0.00	

## DISCUSSION

This study was carried out to determine the effectiveness of peer feedback skill models for the professional development of students. The findings of this study suggest that a significant improvement was observed not only in the perceived feedback skills of the students (Kirkpatrick Level-I) but also in observed feedback skills via workshops (Kirkpatrick Level-II). This is consistent with a recent systematic review that found structured feedback interventions enhance observed performance outcomes as well as learner perception.^15^ In a field with limited comparative research, this study offers proof of the effectiveness of peer feedback interventions in health professions education.^16^

### The immediate impact of the peer feedback skills workshop:

The study used Kirkpatrick Levels-I and II to evaluate the feedback skills workshop. Students’ perceptions of feedback importance (Reaction) and their ability to deliver feedback (Learning) improved, with some evidence of behavioral change, demonstrating that the program met its intended outcomes. These conclusions are further supported by data from comparable Kirkpatrick-based workshop evaluations: participants in structured workshops showed quantifiable learning gains afterward (Level-II) and expressed greater satisfaction and confidence in giving feedback (Level-I).^17^,^18^ The significance of Kirkpatrick Level-I reactions is further supported by recent systematic reviews, which show that student engagement and feedback uptake are highest when learners perceive feedback as pertinent and applicable.^19^

### The intermediate impact of the peer feedback skills workshop:

Next, the enhancement in the feedback skills (learning gain) of the workshop participants was evaluated under Kirkpatrick Level-II using a questionnaire on 16 items already developed by Yorkshire Deanery University.^20^ The same feedback form was provided before and after the workshop to the same group of people. The six-week gap between pre- and post-assessment lessened the likelihood that the participants remembered the choice they had selected in the pre-workshop questionnaire. Methodologically, the spacing between pre/post assessments is consistent with recommendations to minimize response recall bias.^21^ Nevertheless, all the participants responded positively to feedback Performa and supported conducting these workshops for students’ professional development skills.

The results obtained for Coach2coach training demonstrate that all themes in the pre-and post-workshop questionnaires showed an upward trend. The “ability to diagnose patient problems” was rated the least favorite item in R2C2 training pre-workshop findings, with a median score of 2.00 ± 1.00, like the Coach2coach model. However, the improvement in the median value postworkshop for R2C2 was 3.00 ± 1.00, which was smaller than 4.00 ± 3.00 for Coach2coach. This depicts the efficiency of the Coach2coach model for improving peer feedback in this questionnaire item, also validated by p-values.

Mini-PAT data were non-normally distributed (Shapiro-Wilk < 0.05), so the Wilcoxon test was used. Results showed highly significant improvements (p < 0.001) in participants’ feedback post- Coach2coach and R2C2 training, indicating the workshop’s successful outcome.

The R2C2 model mirrored the Coach2coach, showing overall positive improvements in participant feedback. Median scores increased from pre- to post-workshop, with the largest change in “Accessibility/reliability” (3.00 → 4.00, p = 0.000). Improvements were seen across all items except two (items 3 and 8), which were not statistically significant (p > 0.05).

Several authors have considered Coach2coach and R2C2 models to evaluate their effectiveness for peer feedback. For instance, Orr et al^22^ introduced a novel approach to training faculty members in the field of medicine. In this approach, the participants were requested to take part in educational coaching by observing, assessing, and providing comments on various tasks and activities. Conversely, Lockyer et al.^23^ used the R2C2 model to impart feedback and coaching skills among supervisors. Sargeant et al.^24^ used a structured framework to test the same model on sixty one residents and five supervisors for goal-oriented feedback. Recent research on the R2C2 model has also highlighted its theoretical strengths and areas for practical improvement, with an emphasis on maximizing co-creation of action plans and learner-preceptor engagement.^25^ However, systematic reviews of peer feedback practices emphasize that while peer feedback can support professionalism and collaborative learning, more rigorous studies with standardized training and evaluation are needed to better understand implementation and long-term effects.

The pre-post workshop learning gains on both the global rating and the checklist rating scales were highly significant (more than 60 and 35% of learning gain) respectively. Hence, conducting these workshops has a significant positive impact on the medical field. In conclusion, although both models increased students’ peer feedback abilities, allowing them to grow professionally, the Coach2coach model was deemed more efficient in this study. This study’s quasi-experimental design with pre-post assessment, comparison of Coach2coach and R2C2 models, use of a standardized Mini-PAT and a six-week interval to reduce recall bias, are key strengths. These features provide robust evidence on the efficacy of structured peer feedback interventions^26^,^27^ Interactive workshops with role-plays enhanced engagement and practical application, while evaluating both learner perceptions (Kirkpatrick Level-I) and learning outcomes (Level-II) ensures a comprehensive assessment of immediate impact.^28^ Using multi-institutional studies and standardized workshop protocols to improve generalizability and replicability, future research should evaluate long-term behavioral changes (Level-III), institutional/professional outcomes (Level-IV), and the transfer of feedback skills to clinical practice.

### Limitations:

It include its small sample size (52 dental students), which limits its generalizability, its exclusion of students from other healthcare programs, and its focus on final-year students rather than early-year students.

## CONCLUSION

In conclusion, students’ peer feedback skills were greatly enhanced by both COACH2COACH and R2C2 workshops, improving professional development, confidence, and teamwork. However, COACH2COACH proved to be more successful and efficient.

### Authors’ Contribution:

**SS:** Conceived, designed and did data collection.

**UM:** Editing of manuscript, Critical Review, is responsible for integrity of research.

**SMJ:** Literature search**,** statistical analysis and wrote Results.

**SS:** Literature search, critical review and final approval of manuscript.

***Grant Support & Financial Disclosures:*** None.
